# Supplementary material for: Host Plant Modulated Physio-Biochemical Process Enhances Adaptive Response of Sandalwood (Santalum album L.) under Salinity Stress
Source: Plants (Basel). 2024 Apr 22;13(8):1162. doi: 10.3390/plants13081162 (PMC11054670; doi:10.3390/plants13081162)
Supplement: Supplementary file 1 [file plants-13-01162-s001.zip › Supplementary File S2.pdf]

**Methodological procedure in Detail**

## 1) Chlorophyll content

The chlorophyll content was estimated according to the method of Richardson [73].

## 2) Relative water content (%)

The relative water content (RWC) was measured using the method given by Barrs and Weatherley [77]. For its determination, the 4-5 sandalwood leaves were collected and sealed in polythene bags and transported to the laboratory as quickly as possible to minimize the water losses due to evaporation. Samples of leaves were excised, weighed immediately and placed in petriplates containing about 20 ml of distilled water at constant temperature in diffused light for 6 hours. When the leaves turned fully turgid, they were removed from the petri plates, and the adherent water was wiped out with rough filter paper, and the samples were re-weighed for turgid weight. The samples were then kept in an oven for drying (at 70°C for 48 hours) to record the dry weight. These three weights were used to calculate the RWC (%) of leaves according to the formula given by below.

$$RWC(\%) = \frac{Fresh\ weight - Dry\ weight}{Turgid\ weight - Dry\ weight} x\ 100$$

## 3) Relative stress injury (% injury)

The membrane stability was assessed by following the procedure described by Dionisio-Sese and Tobita [79]. For this, the (8-10) leaf discs of 1 cm diameter were kept in 20 ml vials containing 10 ml deionized water at 25°C. After five hours, the electrical conductivity (EC) of the surrounding solution was measured in EC meter (Eutech, India) and was labelled as $EC_a$. Then the samples were kept in a boiling water bath for 50 min to achieve the total killing of tissue. After cooling, the EC of the solution was again measured and designated as $EC_b$. The relative stress injury (RSI) was calculated as per the formula given below and expressed in percentage.

$$RSI\ (\%) = \frac{EC_a}{EC_a + EC_b}$$

## 4) Water Potential (ψw)

For determining the water potential (ψw), the fresh leaves (1 gram) were taken and finely chopped, and the water potential (ψw) was measured using a WP4C Dewpoint Potentiometer (METER Group, Inc. USA). The instrument works on the chilled mirror dew point technique and displays water potential as –MPa. The instrument was calibrated using potassium chloride solutions.

**5) Osmotic Potential (ψs)**

The method described by Kaur *et al.* [76] was followed to measure the osmolality (c) by using the Vapor Pressure Osmometer (Model 5600, ELITech Group, Belgium). For this, the fresh leaves (1 gram) were collected and frozen at -20ºC. The leaves were crushed and the squeezed 5 µl sap extract was used to measure the osmolality. The instrument was calibrated using the osmolality reference standards of sodium chloride (WescorInc, USA). The osmolality reading (mmol $kg^{-1}$) displayed on the digital meter was recorded and converted to osmotic potential by applying the Vant'Haff equation.

$$\psi s \text{ (MPa)} = -c \times 2.58 \times 10\text{-}3$$

**6) Gas Exchange parameters**

The LI-6800 portable photosynthesis system with a standard 6-$cm^2$ leaf chamber (LI-COR, Inc., Lincoln, NE, USA) was used for measuring the photosynthesis (P*n*, µmol $CO_2$ $m^{-2}$ $s^{-1}$), and transpiration rates (E, mmol $H_2O$ $m^{-2}$ $s^{-1}$) between 09:00 am to 11:00 am on two consecutive sunny days. Cuvette conditions were maintained at a photosynthetic photon flux density (PPFD) of 1000 µmol $m^{-2}s^{-1}$, relative humidity >60%, ambient $CO_2$ concentration of 400 ppm and leaf temperature of 25°C [75]. To ensure a similar leaf age and development, the third, fourth and fifth fully expanded leaves of each sandalwood plant were considered for recording these parameters.

**Biochemical parameters of *Santalum album***

**Preparation of enzyme extract**

The extraction buffer for peroxidase enzyme includes 0.01M phosphate buffer (pH 7.0) with 1mM EDTA and 2 percent (w/v) PVP. The extraction medium used for enzymes, ascorbate peroxidase, catalase, and glutathione reductase were made of 0.1M potassium phosphate buffer (pH 7.5) containing 5 percent (w/v) PVP, 1mM EDTA, and 10mM β-mercaptoethanol. The superoxide dismutase enzyme was extracted with buffer containing 0.1M Tris Base buffer (pH 7.5) with 1mM EDTA, glass powder and 2 percent (w/v) PVP. The enzymes were extracted by macerating 2 gm of tissue in 10 ml of ice-cold extraction medium with a pre-chilled mortar and pestle. The homogenate was filtered thoroughly and the filtrate was centrifuged at 10000 rpm at 4ºC for 20 min in a refrigerated centrifuge. The supernatant was collected carefully and used as a crude enzyme preparation.

**7) Superoxide dismutase (SOD)**

Superoxide dismutase was assayed by measuring its ability to inhibit the photochemical reduction of nitroblue tetrazolium (NBT) by adopting the method of Beauchamp and Fridovich,

[83]. The reaction mixture (3ml) contained 60 mM Tris-Base buffer (pH 7.8), 420 mM L-methionine, 90 μM riboflavin, 1.80 mM Nitro blue tetrazolium (NBT), 3.0 mM EDTA, 100 μl of enzyme extract and riboflavin was added at the end. The tubes were thoroughly shaken before being placed 30 cm below the light source, which consisted of two 20 W fluorescent lamps (Phillips, India). The reaction was started by turning on the light and ended after 40 minutes by switching the lights off. After the reaction was completed, the tubes were covered in black cloth to protect them from light.

The control was a non-irradiated reaction mixture that did not develop any colour, and its absorbance at 560 nm was subtracted from the test readings. The reaction mixture with no enzyme extract produced the most colour, while the absorbance decreased with the addition of the enzyme. At 560 nm, the absorbance was recorded and percent inhibition was calculated using the formula:

$$Per\ cent\ inhibition = \frac{V - v}{V}\ x\ 100$$

Where,

V = Absorbance in SOD enzyme absence

v = Absorbance in SOD enzyme presence

One enzyme unit is defined as the amount of enzyme that can inhibit the photochemical reduction of Nitro blue tetrazolium by 50 percent. However, for the specific purpose of kinetic and regulatory properties, enzyme activity was expressed in unit $ml^{-1}$ and calculated using the formula.

$$SOD\ (Units\ ml^{-1})\ = \frac{V - v}{V}\ x\ dillutionfactor$$

**8) Peroxidase (POX)**

The peroxidase was assayed according to the method described by Dias and Costa [84]. The reaction mixture (3ml) contained 2.225 ml of 10 mM potassium phosphate buffer (pH 7.0), 25 μl enzyme extract 0.6 ml guaiacol (1%; v/v) in an aqueous solution. The reaction was started by adding 0.15 ml of 100 mM $H_2O_2$ and an increase in optical density was recorded at 470 nm against a reagent blank without $H_2O_2$. The change in O.D. was recorded at an interval of 15 seconds for 2 min. The linear initial reaction rate was used to determine the activity which was expressed by the guaiacol dehydrogenation product formed per min using the extinction coefficient of 26.6 $mM^{-1}$ $cm^{-1}$.

**9) Catalase (CAT)**

The catalase activity was measured according to the method described by Aebi [85]. The reaction mixture (3ml) contained 100 mM phosphate buffer (pH 7.0), 2 mM $H_2O_2$,1 mM EDTA. The reaction was initiated by the addition of 50µl enzyme extract. The enzyme activity was determined by following the consumption of $H_2O_2$ at 240 nm for 2 min using an extinction coefficient of 39.4 mmol$^{-1}$ cm$^{-1}$. The activity was expressed as nmol $H_2O_2$ consumed min$^{-1}$ g$^{-1}$ fresh weight.

**10) Ascorbate peroxidase (APX)**

Ascorbate peroxidase was assayed by the procedure of Nakano and Asada [86]. The reaction mixture (3ml) contained 90 mM potassium phosphate buffer (pH 7.0), 2 mM ascorbate and 2 mM $H_2O_2$. The reaction was initiated by the addition of 100µl enzyme extract. The decrease in absorbance was measured spectro-photometrically at 290 nm, which corresponds to ascorbic acid oxidation. The enzyme activity was calculated using ascorbate's extinction coefficient of 2.8 mM$^{-1}$ cm$^{-1}$. Enzyme activity was measured in nmol ascorbate decomposed min$^{-1}$g$^{-1}$ FW.

**11) Glutathione reductase (GR)**

Glutathione reductase was assayed by using the method of Halliwell and Foyer [81]. The reaction mixture (3ml) contained 2.4 ml of 0.1 M phosphate buffer (pH 7.5), 200 µl of 0.1 mM EDTA, 100 µl enzyme extract, 200 µl of 3mM oxidized glutathione and 100 µl of 0.2mM NADPH. The addition of NADPH initiated the reaction, and the decrease in absorbance at 340 nm was used to measure the activity. The amount of NADPH oxidised, which corresponded to glutathione reductase activity, was calculated using an extinction coefficient value of 6.12 mM$^{-1}$ cm$^{-1}$ for NADPH. The activity of the enzyme was expressed as nmol NADPH oxidised min$^{-1}$g$^{-1}$ FW (Figure S1).

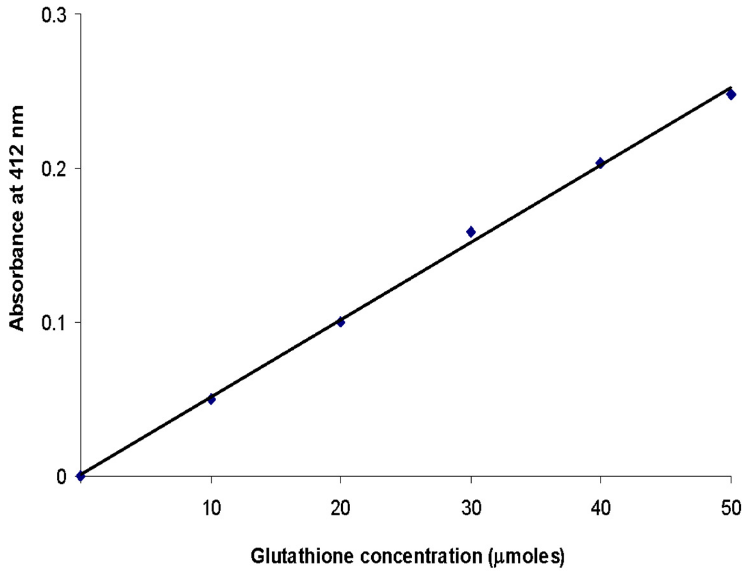


**Figure S1. Standard for glutathione content**

**12) Malondialdehyde (MDA)**

The level of lipid peroxidation was measured in terms of malondialdehyde by using thiobarbituric acid (TBA) reaction with little modification of the method reported by Heath and Packer [80]

**a) Extraction**

One g tissue was homogenized in 5 ml of 0.1 percent TCA (trichloroacetic acid; w/v), the homogenate was then centrifuged at 10000 rpm for 20 min in a refrigerated centrifuge. The supernatant thus obtained was used for the estimation of malondialdehyde content.

**b) Estimation**

In a test tube,1 ml of the supernatant was added with 2 ml of 20 % (w/v) TCA containing 0.5 percent (w/v) 2-thiobarbituric acid (TBA). The mixture was heated in a boiling hot water bath (95ºC) for 30 min with constant stirring and cooled quickly in an ice bath. The absorbance of the supernatant against distilled water was measured at 532 nm, and the value for non-specific absorption at 600 nm was subtracted. The malondialdehyde concentration was calculated using the molar extinction coefficient of 155 $mM^{-1}$ $cm^{-1}$.

**13) Carotenoids**

The amount of carotenoids was estimated by the method proposed by Wellburn [74].

**a) Extraction and Estimation**

To extract carotenoids, 200 mg of fresh leaves were chopped and placed in test tubes containing 10 ml of 80% acetone by incubating overnight. Absorbance at 480 nm, 645 nm, and 663 nm, respectively were taken with a UV-VIS spectrophotometer (SPECORD 210 PLUS) to determine the carotenoids extracted from the acetone [74].

**14) Ionic concentration**

To determine the ionic concentration, the leaves of plant samples were oven dried at 60°C. Dried samples were then grounded and homogenized to a fine powder using a pestle and mortar, and the 0.1 g sample was placed in a 25 mL conical flask. Further, 10 ml di-acidic mixture ($HNO_3$:$HClO_4$, 3:1) was added to a flask and kept overnight. After 24 hours, digestion was done till the formation of white fumes. Further filtration and final volume make-up was done. The content of sodium and potassium in plant parts was measured by recording the observations under a flame photometer. For calcium and magnesium estimation, the reading of digested sample (5 ml) was recorded in an atomic absorption spectrophotometer.
