# Supplementary material for: Host Plant Modulated Physio-Biochemical Process Enhances Adaptive Response of Sandalwood (Santalum album L.) under Salinity Stress
Source: Plants (Basel). 2024 Apr 22;13(8):1162. doi: 10.3390/plants13081162 (PMC11054670; doi:10.3390/plants13081162)
Supplement: Supplementary file 1 [file plants-13-01162-s001.zip › Supplymentary File S1.pdf]

Table S1. Effect of various hosts on diameter and biomass of sandalwood under salinity stress.

Salinity/ Host species	Diameter (mm)					Biomass (g)				
	**Control**	**EC$_{iw}$ 3.0 dS m^{-1}**	**EC$_{iw}$ 6.0 dS m^{-1}**	**EC$_{iw}$ 9.0 dS m^{-1}**	**Mean**	**Control**	**EC$_{iw}$ 3.0 dS m^{-1}**	**EC$_{iw}$ 6.0 dS m^{-1}**	**EC$_{iw}$ 9.0 dS m^{-1}**	**Mean**
M. dubia	8.03	7.21	6.64	5.98	6.96^{a}	154.30	123.39	89.51	64.37	107.89^{a}
C. equisetifolia	6.21	5.82	4.08	3.37	4.87^{d}	135.27	92.67	69.48	44.54	85.49^{c}
C. aurantium	6.51	5.98	5.52	4.36	5.59^{b}	113.99	86.98	58.52	32.49	72.99^{d}
P. emblica	5.97	5.17	4.74	4.08	4.99^{d}	84.35	69.59	47.40	28.55	57.47^{f}
S. cumini	5.35	5.07	4.36	3.74	4.63^{e}	90.86	60.11	50.49	28.02	57.37^{f}
A. ampliceps	5.14	4.77	4.26	3.43	4.4^{fg}	104.37	78.91	42.51	24.14	62.48^{e}
P. granatum	5.19	4.88	4.03	3.59	4.42^{f}	66.43	54.76	34.76	21.07	44.25^{g}
A. indica	6.03	5.85	5.06	4.62	5.39^{c}	117.62	102.4	84.25	50.88	88.79^{b}
L. leucocephala	4.97	4.50	4.02	3.41	4.23^{g}	74.98	49.82	38.2	20.32	45.83^{g}
D. sissoo	7.90	6.96	6.58	6.04	6.87^{a}	145.33	122.07	96.02	65.98	107.35^{a}
Mean	6.13^{a}	5.62^{b}	4.93^{c}	4.26^{d}		108.75^{a}	84.07^{b}	61.11^{c}	38.04^{d}	
HSD$_{0.05}$(Host)	0.19					2.86				
HSD$_{0.05}$(Salinity)	0.11					1.56				
HSD$_{0.05}$(Host × Salinity)	0.46					7.11				

The values carrying different alphabetical superscripts ($^{a,b,c,d,...}$) within the columns above, differ significantly amongst themselves ($p<0.05$). Values are mean ± standard deviations.

Table S2. Effect of various hosts on water and osmotic potential of sandalwood under salinity stress.

**Salinity/ Host species**	**Water potential (MPa)**					**Osmatic potential (MPa)**					**Relative water content (%)**				
	**Control**	**EC_{iw} 3.0 dS m^{-1}**	**EC_{iw} 6.0 dS m^{-1}**	**EC_{iw} 9.0 dS m^{-1}**	**Mean**	**Control**	**EC_{iw} 3.0 dS m^{-1}**	**EC_{iw} 6.0 dS m^{-1}**	**EC_{iw} 9.0 dS m^{-1}**	**Mean**	**Control**	**EC_{iw} 3.0 dS m^{-1}**	**EC_{iw} 6.0 dS m^{-1}**	**EC_{iw} 9.0 dS m^{-1}**	**Mean**
M. dubia	-1.31	-1.64	-2.06	-2.92	-1.98^{a}	-1.53	-1.91	-2.81	-3.54	-2.45^{a}	75.93	72.30	68.59	64.14	70.24^{abcd}
C. equisetifolia	-1.54	-2.36	-2.99	-3.64	-2.63^{c}	-1.67	-2.62	-3.64	-4.36	-3.07^{de}	78.85	76.29	67.02	62.75	71.23^{abc}
C. aurantium	-1.11	-2.07	-2.80	-3.61	-2.40^{bc}	-1.47	-2.57	-3.61	-4.32	-2.99^{cd}	80.15	76.83	65.50	53.44	68.98^{cde}
P. emblica	-1.35	-1.98	-2.70	-3.54	-2.39^{bc}	-1.71	-2.21	-3.36	-4.46	-2.94^{c}	75.13	70.46	65.89	57.67	67.29^{e}
S. cumini	-1.42	-2.14	-3.26	-3.81	-2.66^{c}	-1.85	-2.52	-3.86	-4.63	-3.22^{f}	77.26	70.96	69.22	54.13	67.89^{de}
A. ampliceps	-1.26	-2.16	-2.61	-3.08	-2.28^{ab}	-1.49	-2.51	-3.40	-3.66	-2.77^{b}	74.05	73.73	71.68	58.62	69.52^{bcde}
P. granatum	-1.51	-2.27	-2.65	-3.39	-2.46^{bc}	-1.94	-2.61	-3.36	-4.11	-3.01^{cd}	78.60	78.01	76.25	58.85	72.93^{a}
A. indica	-1.44	-1.98	-2.48	-2.96	-2.22^{ab}	-1.71	-2.16	-3.21	-3.58	-2.67^{b}	77.59	74.70	71.22	60.68	71.05^{abc}
L. leucocephala	-1.46	-2.26	-2.62	-3.32	-2.42^{bc}	-1.90	-2.89	-3.60	-4.24	-3.16^{ef}	78.83	78.06	69.79	55.29	70.49^{abcd}
D. sissoo	-1.55	-2.08	-2.46	-3.12	-2.30^{b}	-1.81	-2.31	-3.17	-3.66	-2.74^{b}	74.86	74.39	71.23	66.56	71.76^{ab}
Mean	-1.40^{a}	-2.09^{b}	-2.66^{c}	-3.34^{d}		-1.71^{a}	-2.43^{b}	-3.40^{c}	-4.06^{d}		77.12	74.57	69.64	59.21	
HSD$_{0.05}$(Host)	0.31					0.12					2.76				
HSD$_{0.05}$(Salinity)	0.18					0.15					1.16				
HSD$_{0.05}$(Host × Salinity)	0.78					0.30					6.87				

The values carrying different alphabetical superscripts ($^{a,b,c,d,\ldots}$) within the columns above, differ significantly amongst themselves ($p<0.05$). Values are mean ± standard deviations.

Table S3. Sodium content of sandalwood grown with different host at variable salinity levels.

Host species	Salinity Treatment				
	Control	EC_{iw} 3.0 dS m^{-1}	EC_{iw} 6.0 dS m^{-1}	EC_{iw} 9.0 dS m^{-1}	Mean
M. dubia	1.22	1.46	1.82	2.23	1.68^{e}
C. equisetifolia	1.37	1.68	1.96	2.52	1.88^{c}
C. aurantium	1.41	1.74	2.04	2.65	1.96^{b}
P. emblica	1.35	1.68	1.98	2.59	1.90^{bc}
S. cumini	1.48	1.84	2.28	2.76	2.09^{a}
A. ampliceps	1.25	1.53	1.94	2.37	1.77^{d}
P. granatum	1.34	1.65	2.12	2.58	1.92^{bc}
A. indica	1.24	1.48	1.83	2.31	1.72^{de}
L. leucocephala	1.28	1.59	1.85	2.36	1.77^{d}
D. sissoo	1.23	1.45	1.70	2.03	1.60^{f}
**Mean**	1.32^{d}	1.61^{c}	1.95^{b}	2.44^{a}	
**$HSD_{0.05}$(Host)**	0.07				
**$HSD_{0.05}$(Salinity)**	0.04				
**$HSD_{0.05}$(Host × Salinity)**	0.16				

The values carrying different alphabetical superscripts ($^{a,b,c,d,...}$) within the columns above, differ significantly amongst themselves ($p<0.05$). Values are mean ± standard deviations.

Table S4. Potassium content of sandalwood grown with different host at variable salinity levels.

Host species	Salinity Treatment				
	Control	EC_{iw} 3.0 dS m^{-1}	EC_{iw} 6.0 dS m^{-1}	EC_{iw} 9.0 dS m^{-1}	Mean
M. dubia	2.65	2.51	2.35	1.98	2.37^{a}
C. equisetifolia	2.58	2.40	2.14	1.83	2.24^{b}
C. aurantium	2.52	2.38	2.05	1.71	2.16^{c}
P. emblica	2.46	2.31	1.98	1.62	2.09^{d}
S. cumini	2.47	2.25	2.08	1.74	2.13^{cd}
A. ampliceps	2.38	2.23	1.99	1.79	2.10^{cd}
P. granatum	2.36	2.13	1.85	1.69	2.01^{e}
A. indica	2.60	2.47	2.25	1.99	2.33^{a}
L. leucocephala	2.39	2.22	2.04	1.73	2.09^{d}
D. sissoo	2.62	2.50	2.37	1.99	2.37^{a}
**Mean**	2.50^{a}	2.34^{b}	2.11^{c}	1.81^{d}	
**$HSD_{0.05}$(Host)**	0.07				
**$HSD_{0.05}$(Salinity)**	0.05				
**$HSD_{0.05}$(Host × Salinity)**	0.17				

The values carrying different alphabetical superscripts ($^{a,b,c,d,\ldots}$) within the columns above, differ significantly amongst themselves ($p<0.05$). Values are mean ± standard deviations.

Table S5. Calcium content of sandalwood grown with different host at variable salinity levels.

Host species	Salinity Treatment				
	Control	EC_{iw} 3.0 dS m^{-1}	EC_{iw} 6.0 dS m^{-1}	EC_{iw} 9.0 dS m^{-1}	Mean
M. dubia	1.80	1.68	1.57	1.47	1.63[a]
C. equisetifolia	1.76	1.61	1.44	1.28	1.52[c]
C. aurantium	1.53	1.38	1.22	1.07	1.30[d]
P. emblica	1.32	1.19	1.05	0.92	1.12[g]
S. cumini	1.46	1.32	1.16	1.02	1.24[e]
A. ampliceps	1.17	1.06	0.94	0.82	1.00[h]
P. granatum	1.39	1.26	1.12	0.97	1.19[f]
A. indica	1.68	1.56	1.45	1.32	1.50[c]
L. leucocephala	1.45	1.31	1.16	1.01	1.23[e]
D. sissoo	1.69	1.62	1.55	1.44	1.58[b]
**Mean**	1.53[a]	1.40[b]	1.27[c]	1.13[d]	
**$HSD_{0.05}$(Host)**	0.04				
**$HSD_{0.05}$(Salinity)**	0.02				
**$HSD_{0.05}$(Host × Salinity)**	0.11				

The values carrying different alphabetical superscripts ([a,b,c,d,…]) within the columns above, differ significantly amongst themselves ($p<0.05$). Values are mean ± standard deviations.

Table S6. Magnesium content of sandalwood grown with different host at variable salinity levels.

Host species	Salinity Treatment				
	Control	EC_{iw} 3.0 dS m^{-1}	EC_{iw} 6.0 dS m^{-1}	EC_{iw} 9.0 dS m^{-1}	Mean
M. dubia	0.92	0.90	0.87	0.84	0.88[a]
C. equisetifolia	0.83	0.81	0.78	0.74	0.79[b]
C. aurantium	0.77	0.74	0.71	0.67	0.72[c]
P. emblica	0.64	0.61	0.59	0.55	0.60[e]
S. cumini	0.73	0.70	0.67	0.64	0.69[d]
A. ampliceps	0.66	0.64	0.61	0.57	0.62[fg]
P. granatum	0.70	0.67	0.64	0.60	0.65[e]
A. indica	0.81	0.79	0.76	0.73	0.77[b]
L. leucocephala	0.68	0.66	0.63	0.59	0.64[ef]
D. sissoo	0.89	0.88	0.87	0.85	0.87[a]
**Mean**	0.76[a]	0.74[b]	0.71[c]	0.68[d]	
**$HSD_{0.05}$(Host)**	0.03				
**$HSD_{0.05}$(Salinity)**	0.02				
**$HSD_{0.05}$(Host × Salinity)**	0.07				

The values carrying different alphabetical superscripts ([a,b,c,d,…]) within the columns above, differ significantly amongst themselves ($p<0.05$). Values are mean ± standard deviations.

Table S7. Membrane injury of sandalwood grown with different host at variable salinity levels.

Host species	Salinity Treatment				
	**Control**	**EC_{iw} 3.0 dS m^{-1}**	**EC_{iw} 6.0 dS m^{-1}**	**EC_{iw} 9.0 dS m^{-1}**	**Mean**
M. dubia	10.85	18.49	28.71	31.42	22.37^{f}
C. equisetifolia	13.31	26.76	33.19	39.67	28.23^{cd}
C. aurantium	13.96	28.29	36.35	41.95	30.14^{a}
P. emblica	12.02	25.27	34.15	38.32	27.44^{d}
S. cumini	14.38	23.07	34.21	43.99	28.91^{bc}
A. ampliceps	13.09	16.15	28.28	36.68	23.55^{e}
P. granatum	12.17	25.97	38.37	42.62	29.78^{ab}
A. indica	13.07	23.36	34.91	38.38	27.43^{d}
L. leucocephala	12.85	26.43	35.82	40.93	29.01^{abc}
D. sissoo	11.25	17.37	24.22	33.03	21.47^{f}
**Mean**	12.70^{d}	23.11^{c}	32.82^{b}	38.70^{a}	
**$HSD_{0.05}$(Host)**	1.15				
**$HSD_{0.05}$(Salinity)**	0.57				
**$HSD_{0.05}$(Host × Salinity)**	2.87				

The values carrying different alphabetical superscripts ($^{a,b,c,d,\ldots}$) within the columns above, differ significantly amongst themselves ($p<0.05$). Values are mean ± standard deviations.

Table S8. Malondialdehyde (MDA) content of sandalwood grown with different host at variable salinity levels.

Host species	Salinity Treatment				
	**Control**	**EC_{iw} 3.0 dS m^{-1}**	**EC_{iw} 6.0 dS m^{-1}**	**EC_{iw} 9.0 dS m^{-1}**	**Mean**
M. dubia	0.97	0.98	1.03	1.24	1.05^{a}
C. equisetifolia	0.86	0.96	0.97	1.20	1.00^{bc}
C. aurantium	0.79	0.90	1.07	1.14	0.98^{cd}
P. emblica	0.90	0.96	1.02	1.19	1.02^{ab}
S. cumini	0.86	0.96	1.05	1.19	1.02^{b}
A. ampliceps	0.79	0.82	0.95	0.99	0.89^{g}
P. granatum	0.81	0.90	0.98	1.10	0.95^{de}
A. indica	0.80	0.86	0.90	1.05	0.90^{fg}
L. leucocephala	0.80	0.86	0.95	1.07	0.92^{ef}
D. sissoo	0.81	0.85	0.89	0.97	0.88^{g}
**Mean**	0.84^{d}	0.90^{c}	0.98^{b}	1.11^{a}	
**$HSD_{0.05}$(Host)**	0.033				
**$HSD_{0.05}$(Salinity)**	0.019				
**$HSD_{0.05}$(Host × Salinity)**	0.082				

The values carrying different alphabetical superscripts ($^{a,b,c,d,\ldots}$) within the columns above, differ significantly amongst themselves ($p<0.05$). Values are mean ± standard deviations.

Table S9. Ascorbate peroxidase (APX) (units g^{-1} FW) activity of sandalwood grown with different host at variable salinity levels.

Host species	Salinity Treatment				
	Control	EC_{iw} 3.0 dS m^{-1}	EC_{iw} 6.0 dS m^{-1}	EC_{iw} 9.0 dS m^{-1}	Mean
M. dubia	0.79	1.69	2.53	3.60	2.15[a]
C. equisetifolia	0.62	0.64	1.23	1.90	1.10[f]
C. aurantium	0.48	0.55	0.89	1.25	0.79[h]
P. emblica	0.48	0.57	1.24	2.29	1.14[f]
S. cumini	0.43	0.49	0.67	1.01	0.65[i]
A. ampliceps	0.74	0.99	1.39	2.46	1.39[d]
P. granatum	0.40	0.59	0.99	1.60	0.89[g]
A. indica	0.66	1.05	1.74	3.10	1.64[c]
L. leucocephala	0.56	0.94	1.43	1.95	1.22[e]
D. sissoo	0.68	1.34	2.06	3.32	1.85[b]
**Mean**	0.58[d]	0.88[c]	1.42[b]	2.25[a]	
**$HSD_{0.05}$(Host)**	0.07				
**$HSD_{0.05}$(Salinity)**	0.03				
**$HSD_{0.05}$(Host × Salinity)**	0.16				

The values carrying different alphabetical superscripts ([a,b,c,d,....]) within the columns above, differ significantly amongst themselves ($p<0.05$). Values are mean ± standard deviations.

Table S10. Catalase (CAT) (units g^{-1} FW) activity of sandalwood grown with different host at variable salinity levels.

Host species	Salinity Treatment				
	Control	EC_{iw} 3.0 dS m^{-1}	EC_{iw} 6.0 dS m^{-1}	EC_{iw} 9.0 dS m^{-1}	Mean
M. dubia	0.14	0.19	0.26	0.36	0.24[b]
C. equisetifolia	0.12	0.15	0.19	0.27	0.18[f]
C. aurantium	0.09	0.14	0.17	0.22	0.16[g]
P. emblica	0.15	0.19	0.22	0.29	0.21[de]
S. cumini	0.10	0.14	0.17	0.24	0.16[g]
A. ampliceps	0.15	0.19	0.27	0.30	0.22[c]
P. granatum	0.11	0.15	0.19	0.27	0.18[f]
A. indica	0.12	0.18	0.24	0.33	0.22[cd]
L. leucocephala	0.13	0.17	0.23	0.30	0.21[e]
D. sissoo	0.14	0.22	0.28	0.37	0.25[a]
**Mean**	0.12[d]	0.17[c]	0.22[b]	0.30[a]	
**$HSD_{0.05}$(Host)**	0.008				
**$HSD_{0.05}$(Salinity)**	0.003				
**$HSD_{0.05}$(Host × Salinity)**	0.020				

The values carrying different alphabetical superscripts ([a,b,c,d,....]) within the columns above, differ significantly amongst themselves ($p<0.05$). Values are mean ± standard deviations.

Table S11. Peroxidase (POX) (units g^{-1} FW) activity of sandalwood grown with different host at variable salinity levels.

Host species	Salinity Treatment				
	Control	EC_{iw} 3.0 dS m^{-1}	EC_{iw} 6.0 dS m^{-1}	EC_{iw} 9.0 dS m^{-1}	Mean
M. dubia	195.58	258.71	304.07	458.86	304.30^{a}
C. equisetifolia	178.11	248.18	298.18	366.17	272.66^{c}
C. aurantium	218.73	261.97	294.34	310.55	271.40^{c}
P. emblica	207.82	235.76	252.66	351.87	262.03^{de}
S. cumini	204.41	241.82	290.74	327.92	266.22^{cd}
A. ampliceps	151.67	179.05	238.60	317.42	221.68^{g}
P. granatum	161.77	227.07	282.37	341.49	253.17^{e}
A. indica	219.44	258.33	343.21	407.89	307.22^{a}
L. leucocephala	155.11	200.29	277.52	318.27	237.80^{f}
D. sissoo	156.87	227.76	369.46	424.16	294.56^{b}
**Mean**	184.95^{d}	233.89^{c}	295.11^{b}	362.46^{a}	
**$HSD_{0.05}$(Host)**	9.04				
**$HSD_{0.05}$(Salinity)**	5.58				
**$HSD_{0.05}$(Host × Salinity)**	22.48				

The values carrying different alphabetical superscripts ($^{a,b,c,d,...}$) within the columns above, differ significantly amongst themselves ($p<0.05$). Values are mean ± standard deviations.

Table S12. Superoxide dismutase (SOD) (units g^{-1} FW) activity of sandalwood grown with different host at variable salinity levels.

Host species	Salinity Treatment				
	Control	EC_{iw} 3.0 dS m^{-1}	EC_{iw} 6.0 dS m^{-1}	EC_{iw} 9.0 dS m^{-1}	Mean
M. dubia	11.54	21.03	31.74	39.63	25.98^{a}
C. equisetifolia	13.55	19.02	25.62	30.01	22.05^{b}
C. aurantium	8.37	9.97	11.01	16.85	11.55^{ef}
P. emblica	6.86	12.93	16.11	23.24	14.78^{d}
S. cumini	7.76	9.62	10.42	15.48	10.82^{f}
A. ampliceps	6.68	9.37	14.18	20.55	12.69^{e}
P. granatum	6.54	7.63	11.76	19.00	11.23^{ef}
A. indica	9.04	17.01	22.97	30.09	19.78^{c}
L. leucocephala	8.15	11.69	17.53	22.43	14.95^{d}
D. sissoo	9.12	16.11	25.44	36.05	21.68^{b}
**Mean**	8.76^{d}	13.44^{c}	18.68^{b}	25.33^{a}	
**$HSD_{0.05}$(Host)**	1.68				
**$HSD_{0.05}$(Salinity)**	2.57				
**$HSD_{0.05}$(Host × Salinity)**					

The values carrying different alphabetical superscripts ($^{a,b,c,d,...}$) within the columns above, differ significantly amongst themselves ($p<0.05$). Values are mean ± standard deviations.

Table S13. Glutathione reductase (GR) (units g-1 FW) activity of sandalwood grown with different host at variable salinity levels.

Host species	Salinity Treatment				
	Control	EC_{iw} 3.0 dS m^{-1}	EC_{iw} 6.0 dS m^{-1}	EC_{iw} 9.0 dS m^{-1}	Mean
M. dubia	1.58	1.75	1.98	2.19	1.88[a]
C. equisetifolia	0.86	0.99	1.11	1.53	1.12[d]
C. aurantium	0.50	0.53	0.55	0.74	0.58[g]
P. emblica	0.54	0.64	0.80	0.93	0.73[f]
S. cumini	0.52	0.64	0.81	0.97	0.73[f]
A. ampliceps	0.70	0.80	1.22	1.64	1.09[d]
P. granatum	1.14	1.17	1.08	1.00	1.10[d]
A. indica	1.41	1.77	1.88	2.10	1.79[b]
L. leucocephala	0.41	0.75	1.04	1.43	0.91[e]
D. sissoo	1.22	1.53	1.97	2.07	1.70[c]
**Mean**	0.89[d]	1.06[c]	1.25[b]	1.46[a]	
**$HSD_{0.05}$(Host)**	0.06				
**$HSD_{0.05}$(Salinity)**	0.04				
**$HSD_{0.05}$(Host × Salinity)**	0.13				

The values carrying different alphabetical superscripts (a,b,c,d,...) within the columns above, differ significantly amongst themselves (p<0.05). Values are mean ± standard deviations.

Table S14a. Host preferential and ranks of the host in control condition estimated through weighted coefficients (β_s).

Host names	Constant (α)	0.82× RWC	(-8.05)× Pn	(-3.62)× K	(-40.27)× NaK	(-90.35)× MDA	64.27× APX	(-0.32)× POX	3.76× SOD	7.75× GR	8.48× WP	17.57× DIA	PBIO	Rank	Actual Bio	Rank
Melia dubia	74.29	62.26	-38.96	-18.35	-10.56	-87.28	50.56	-62.59	43.39	12.25	-11.11	140.97	**154.86**	**1**	**154.3**	**1**
Dalbergia sissoo		61.39	-51.01	-22.58	-8.02	-72.73	43.92	-50.20	34.28	9.51	-13.14	138.86	**144.56**	**2**	**145.33**	**2**
Casuarina equisetifolia		64.66	-28.42	-19.67	-15.96	-77.82	40.06	-56.98	50.95	6.67	-13.06	109.11	**133.83**	**3**	**135.27**	**3**
Azadirachta indica		63.63	-27.58	-17.06	-15.52	-72.37	42.85	-70.22	33.98	10.93	-12.21	105.89	**116.59**	**4**	**117.62**	**4**
Citrus aurantium		65.72	-20.82	-17.86	-17.38	-71.68	31.06	-69.99	31.47	3.82	-9.41	114.44	113.67	5	113.99	5
Acacia ampliceps		60.73	-37.70	-17.22	-13.21	-71.26	47.56	-48.53	25.12	5.48	-10.68	90.37	104.93	6	104.37	6
Syzygium cumini		63.35	-19.91	-17.89	-9.32	-77.70	27.42	-65.41	29.18	4.03	-12.04	93.94	89.94	7	90.86	7
Phyllanthus emblica		61.60	-24.85	-18.68	-14.04	-81.38	31.06	-66.50	25.78	4.19	-11.45	104.83	84.87	8	84.35	8
Leucaena leucocephala		64.64	-57.80	-15.40	-13.47	-72.49	35.99	-49.63	30.66	3.13	-12.38	87.32	74.86	9	74.98	9
Punica granatum		64.45	-52.06	-19.57	-12.27	-73.61	25.49	-51.77	24.59	8.86	-12.80	91.31	66.92	10	66.43	10
		**Model Fitted: Predicted Biomass ~ 74.29+ 0.82 $_{RWC}$+(-8.05)$_{Pn}$+(-3.62)$_{K}$+(-40.57)$_{NaK}$+(-90.35)$_{MDA}$+64.27$_{APX}$+ (-0.32)$_{POX}$+ 3.76$_{SOD}$+ 7.75$_{GR}$+ 8.48$_{WP}$+ 17.57$_{Dia}$: R^2 = 0.98**														

Table S14b. Host preferential and ranks of the host in low salinity stress (EC_{iw}~$3dSm^{-1}$).

Host names	Constant (α)	45.89× Pn	(-8.48)× Chl	(-15.04)× MI	(-1.53)× SPAD	(-2.92)× Na	26.07× K	13.81× MDA	(-81.03)× APX	46.07× SOD	0.73× DIA	P Biomass	Rank	Actual Biomass	Rank
Melia dubia		-34.51	-17.90	-28.29	-52.84	62.22	58.78	-79.60	77.70	15.35	77.22	124.03	1	123.39	1
Casuarina equisetifolia		-23.29	-18.10	-40.94	-38.98	63.18	74.48	-77.44	29.64	13.88	62.33	90.66	4	92.67	4
Citrus aurantium		-13.31	-18.60	-43.28	-35.47	69.87	55.79	-72.66	25.34	7.28	64.08	84.92	5	86.98	5
Phyllanthus emblica		-21.00	-18.15	-38.66	-40.59	66.57	65.51	-77.95	26.11	9.44	55.37	72.53	7	69.59	7
Syzygium cumini		-9.13	-15.29	-35.29	-40.73	53.27	56.53	-77.98	22.73	7.02	54.30	61.31	8	60.11	8
Acacia ampliceps		-31.04	-10.98	-24.71	-46.44	51.88	55.88	-66.66	45.30	6.84	51.05	77.02	6	78.91	6
Punica granatum		-27.14	-6.02	-39.73	-50.22	61.26	57.77	-72.52	27.03	5.57	52.30	54.20	9	54.76	9
Azadirachta indica		-24.79	-9.32	-35.74	-43.99	62.13	55.01	-69.85	48.22	12.42	62.62	102.60	3	102.40	3
Leucaena leucocephala		-44.07	-6.92	-40.43	-42.34	52.23	56.48	-70.09	43.31	8.53	48.23	50.82	10	49.82	10
Dalbergia sissoo		-46.44	-8.22	-26.57	-46.29	50.84	73.10	-68.52	61.58	11.76	74.51	121.62	2	122.07	2
		**Model Fitted: Predicted Biomass ~ 45.890+ (-8.48)$_{Pn}$+ (-15.040)$_{Chl}$+ (-1.530)$_{MI}$+(-2.920)$_{SPAD}$+ 26.070 $_{Na}$+13.810 $_{K}$ +(-81.030)$_{MDA}$+46.070$_{APX}$+ 0.730$_{SOD}$+10.710 $_{Dia}$:** **R^2 = 0.98**													

Table S14c. Host preferential and ranks of the host in moderate salinity stress (EC_{iw}~6 dSm^{-1}).

Host names	Constant (α)	(-5.42) × Pn	(-0.35)× MI	(-1.83)× SPAD	6.63× Na	8.84× K	0.11× POX	30.59× GR	3.05× WP	7.83× DIA	(-0.31) × RWC	P Biomass	Rank	Actual Biomass	Rank
Melia dubia		-20.07	-10.05	-28.27	20.71	34.45	33.45	60.67	-6.28	51.99	-21.26	88.77	2	89.51	2
Casuarina equisetifolia		-10.75	-11.61	-18.39	32.13	35.09	32.80	33.85	-9.12	32.00	-20.78	68.68	4	69.48	4
Citrus aurantium		-6.36	-12.72	-21.69	32.13	28.79	32.38	16.82	-8.54	43.27	-20.31	57.23	5	58.52	5
Phyllanthus emblica		-9.30	-11.95	-23.24	30.63	27.02	27.79	24.57	-8.24	37.11	-20.43	47.42	7	47.40	7
Syzygium cumini		-3.50	-11.97	-26.80	26.98	32.09	31.98	24.78	-9.94	34.11	-21.46	49.71	6	50.49	6
Acacia ampliceps		-12.99	-9.90	-25.07	22.14	27.40	26.25	37.42	-7.96	33.36	-22.22	41.88	8	42.51	8
Punica granatum		-15.39	-13.43	-28.46	26.83	27.17	31.06	33.04	-8.08	31.53	-23.64	34.08	10	34.76	10
Azadirachta indica		-12.43	-12.22	-23.42	22.50	29.64	37.75	57.61	-7.56	39.65	-22.08	82.89	3	84.25	3
Leucaena leucocephala		-20.85	-12.54	-24.43	27.85	30.00	30.53	31.81	-7.99	31.50	-21.63	37.69	9	38.20	9
Dalbergia sissoo		-26.20	-8.48	-24.61	20.07	37.86	40.64	60.16	-7.50	51.50	-22.08	94.81	1	96.02	1
		**Model Fitted: Predicted Biomass ~ -26.550 +(-5.42$_{Pn}$)+ (-0.35)$_{MI}$+(-1.83)$_{SPAD}$ + 6.63$_{Na}$+ 8.84$_{K}$+ 0.11$_{POX}$+ 30.59$_{GR}$+ 3.05$_{WP}$+ 7.83$_{DIA}$+ (-0.31)$_{RWC}$ R^2 = 0.99**													

Table S14d. Host preferential and ranks of the host in higher salinity stress (EC_{iw}~9dSm^{-1}).

Host names	Constant (α)	0.44 × RWC	(-3.08)× E	(-9.29)× Chl	(-0.78)× MI	(-1.51)× SPAD	3.38× Na	5.66× K	27.08× MDA	(-11.11)× APX	62.38 × CAT	15.86 × GR	13.15× DIA	P Biomass	Rank
Melia dubia		28.22	-2.96	-8.70	-24.51	-22.51	11.78	19.45	33.51	-40.00	22.58	34.73	78.58	**64.04**	2
Casuarina equisetifolia		27.61	-1.87	-7.37	-30.94	-16.23	26.88	15.24	32.52	-21.18	16.74	24.21	44.37	**43.84**	4
Citrus aurantium		23.52	-1.62	-8.89	-32.72	-15.22	22.47	10.79	30.84	-13.85	13.47	11.74	57.28	**31.65**	5
Phyllanthus emblica		25.37	-1.93	-9.41	-29.89	-15.33	18.57	13.79	32.34	-25.52	18.36	14.80	53.65	**28.67**	6
Syzygium cumini		23.82	-1.80	-7.77	-34.31	-13.89	16.81	10.40	32.20	-11.18	14.91	15.33	49.18	**27.54**	7
Acacia ampliceps		25.80	-5.78	-5.51	-28.61	-16.46	15.48	15.77	26.85	-27.26	18.55	26.06	45.08	**23.83**	8
Punica granatum		25.89	-5.10	-2.82	-33.25	-19.71	18.08	11.60	29.76	-17.78	16.99	15.75	47.23	**20.53**	9
Azadirachta indica		26.70	-2.87	-3.75	-29.93	-14.50	16.09	16.15	28.48	-34.40	20.59	33.25	60.73	**50.38**	3
Leucaena leucocephala		24.33	-6.06	-3.56	-31.92	-19.03	17.84	11.21	28.99	-21.66	18.63	22.73	44.82	**20.17**	10
Dalbergia sissoo		29.29	-7.85	-4.27	-25.77	-17.84	11.59	21.15	26.38	-36.85	23.37	32.94	79.48	**65.47**	1
		**Model Fitted: Predicted Biomass ~ ~ -66.140 + 0.440$_{RWC}$+ (-3.080)$_{E}$+(-9.290)$_{Chl}$+ (-0.780)$_{MI}$+ (-1.510$_{SPAD}$) + 3.380$_{Na}$+ 5.660$_{K}$+ 27.080$_{MDA}$+ (-11.110)$_{APX}$+ 62.380$_{CAT}$+15.860$_{GR}$+13.150$_{DIA}$ R^2 = 0.99**													

Table S15. Characteristics of host plant species used in the experiment.

**Common name**	**Binomial name**	**Family**	**Wild/ cultivated**	**Plant type**	**Nature**	**Leguminous (N_2 fixing ability)**
*Lal mehndi	*Alternanthera dentata (Moench) Stuchlík ex R. E. Fr.*	Amaranthaceae	Cultivated	Dicot	Evergreen	(❌)
Malabar Neem	*Melia dubia* Cav.	Meliaceae	Cultivated	Dicot	Deciduous	(❌)
#Whistling pine	*Casuarina equisetifolia* L.	Casuarinaceae	Wild	Dicot	Evergreen	(✓)
Nimbu	*Citrus aurantium* L.	Rutaceae	Cultivated	Dicot	Evergreen	(❌)
Aonla	*Phyllanthus emblica* L.	Meliaceae	Cultivated	Dicot	Deciduous	(❌)
Jamun	*Syzygium cumini* (L.) Skeels.	Myrtaceae	Wild	Dicot	Evergreen	(❌)
Salt wattle	*Acacia ampliceps* Maslin	Fabaceae	Wild	Dicot	Evergreen	(✓)
Anar	*Punica granatum* L.	Lythraceae	Cultivated	Dicot	Deciduous	(❌)
Neem	*Azadirachta indica* A. Juss	Meliaceae	Wild	Monocot	Deciduous	(❌)
Subabul	*Leucaena leucocephala* (Lam.) de Wit	Fabaceae	Wild	Dicot	Evergreen	(✓)
Shisham	*Dalbergia sissoo* Roxb.	Fabaceae	Cultivated	Dicot	Deciduous	(✓)

Common Nursery Host, # Fix Nitrogen through Frankia

Table S16. Physico-chemical properties of the soil before conducting the experiment.

**Soil Parameter**	**Values**
pH_2	7.03± 0.47
EC_e (dS m^{-1})	1.49 ± 0.21
Texture	Sandy loam
Organic carbon (g kg^{-1})	0.697±0.02
Available Nitrogen (N) kg ha^{-1}	133.8 ±14.04
Available Phosphorus (P) kg ha^{-1}	33.77±4.28
Available Potassium (K) kg ha^{-1}	358.4±10.59
Na^+ (ppm)	160
Ca^{++} (meqL^{-1})	4.8
Mg^{++} (meqL^{-1})	4.8
Cl^- (meqL^{-1})	1.5
CO_3^- (meqL^{-1})	0.0
HCO_3^- (meqL^{-1})	0.9

Note: Data were expressed as the means of 10 replications and presented as means values ± standard deviation

Table S17. Quality parameters of applied irrigation water.

Properties	Saline water	Best available water
pH_2	8.03 ± 0.47	7.41± 0.51
EC_2 (dS m^{-1})	16.49 ± 0.21	0.72 ± 0.05
Na^+ (meqL^{-1})	162	3.28
K^+ (meqL^{-1})	1.1	0.14
Ca^{++} + Mg^{++} (meqL^{-1})	60	4.2
Cl^- (meqL^{-1})	97	1.3
CO_3^- (meqL^{-1})	1.5	Nil
HCO_3^- (meqL^{-1})	1.8	3.4

Note: Data were expressed as the means of 10 replications and presented as means values ± standard deviation
